# Ionizing Particle Radiation as a Modulator of Endogenous Bone Marrow Cell Reprogramming: Implications for Hematological Cancers

**DOI:** 10.3389/fonc.2015.00231

**Published:** 2015-10-14

**Authors:** Sujatha Muralidharan, Sharath P. Sasi, Maria A. Zuriaga, Karen K. Hirschi, Christopher D. Porada, Matthew A. Coleman, Kenneth X. Walsh, Xinhua Yan, David A. Goukassian

**Affiliations:** ^1^Whitaker Cardiovascular Institute, Boston University School of Medicine, Boston, MA, USA; ^2^Cardiovascular Research Center, GeneSys Research Institute, Boston, MA, USA; ^3^Yale Cardiovascular Research Center, Yale School of Medicine, New Haven, CT, USA; ^4^Wake Forest Institute for Regenerative Medicine, Wake Forest School of Medicine, Winston-Salem, NC, USA; ^5^Radiation Oncology, School of Medicine, University of California Davis, Sacramento, CA, USA; ^6^Lawrence Livermore National Laboratory, Livermore, CA, USA; ^7^Tufts University School of Medicine, Boston, MA, USA

**Keywords:** HSC, progenitors, radiation, endogenous reprogramming, hematological cancer

## Abstract

Exposure of individuals to ionizing radiation (IR), as in the case of astronauts exploring space or radiotherapy cancer patients, increases their risk of developing secondary cancers and other health-related problems. Bone marrow (BM), the site in the body where hematopoietic stem cell (HSC) self-renewal and differentiation to mature blood cells occurs, is extremely sensitive to low-dose IR, including irradiation by high-charge and high-energy particles. Low-dose IR induces DNA damage and persistent oxidative stress in the BM hematopoietic cells. Inefficient DNA repair processes in HSC and early hematopoietic progenitors can lead to an accumulation of mutations whereas long-lasting oxidative stress can impair hematopoiesis itself, thereby causing long-term damage to hematopoietic cells in the BM niche. We report here that low-dose ^1^H- and ^56^Fe-IR significantly decreased the hematopoietic early and late multipotent progenitor (E- and L-MPP, respectively) cell numbers in mouse BM over a period of up to 10 months after exposure. Both ^1^H- and ^56^Fe-IR increased the expression of pluripotent stem cell markers *Sox2*, *Nanog*, and *Oct4* in L-MPPs and 10 months post-IR exposure. We postulate that low doses of ^1^H- and ^56^Fe-IR may induce endogenous cellular reprogramming of BM hematopoietic progenitor cells to assume a more primitive pluripotent phenotype and that IR-induced oxidative DNA damage may lead to mutations in these BM progenitors. This could then be propagated to successive cell lineages. Persistent impairment of BM progenitor cell populations can disrupt hematopoietic homeostasis and lead to hematologic disorders, and these findings warrant further mechanistic studies into the effects of low-dose IR on the functional capacity of BM-derived hematopoietic cells including their self-renewal and pluripotency.

## Introduction

Exposure to ionizing radiation (IR), specifically high-energy protons (^1^H) and ions with high charge and high energy (HZE particles), is one of the major risks during spaceflight beyond low Earth orbit (LEO) (1, 2). For example, astronauts on future Mars missions are expected to encounter ~0.6 Sv of IR during 180 days transit to Mars (3). In this case, it is estimated that each cell in an astronaut’s body will be traversed by a low-dose ^1^H every 3–4 days, helium nuclei every few weeks, and HZE particles, such as iron (^56^Fe), every few months. The radiation encountered by astronauts in LEO in proximity of the van Allen belt is mostly from ^1^H particles from solar winds, trapped in the earth’s magnetic field (4). This type of low linear energy transfer (LET) radiation, including γ rays and X-rays, deposit relatively little energy as they pass through matter. However, venturing beyond the van Allen belt and into deep space, astronauts will encounter a significant amount of galactic cosmic radiation which contains not only high-energy ^1^H and alpha particles but also high-LET radiation from HZE particles, such as ^56^Fe and ^28^Si (4). These high-LET HZE ions have a greater propensity for ionization and they deposit large amounts of energy along their tracks; and thus have greater potential for causing damage to tissues. These types of low- and high-LET radiation are also encountered on earth. For example, low energy ^1^H and HZE carbon ion IR are being used in cancer radiotherapy regimens for patients suffering from breast cancer, esophageal cancer, adenocarcinoma, and hepatocellular carcinoma (5–10). To date, the biological effects of low-dose ^1^H and HZE ion IR have not been fully investigated.

Radiation dose is an important factor for consideration in the biological effects of low- and high-LET radiation. Although epidemiological studies based on atomic bomb survivors and cancer radiotherapy patients have provided insight into the biological effects of moderate to high doses of IR (11, 12), the effects of low-dose IR over long periods of time remain to be elucidated. A single high dose of radiation may induce significant tissue and cell damage; however, the biological effects of low-dose IR may be more relevant in disease processes, owing to IR-induced aberrations at the genetic or epigenetic levels. This “reprogramming” can be propagated in surviving cells and can have long-term implications in the health of the IR exposed individual.

This article focuses on the biological relevance of low-dose low-LET ^1^H and high-LET HZE ^56^Fe radiation. Charged ^1^H particles are the most abundant radiation found in deep space and HZE particles (1% of galactic cosmic rays) contribute to more than 40% of the equivalent dose exposure for the astronauts (4, 13, 14). Notably, low-energy ^1^H particles are also being used as a source of radiation for the treatment of cancers owing to their favorable radiation dose distribution in cancerous tissue (15, 16). Therefore, studying the biological consequences of these types of radiation is of significance for understanding the consequences of both space missions and cancer therapy regimens.

## Effects of Ionizing Radiation on the Bone Marrow

### Radiation-Induced DNA Damage and Oxidative Stress in BM Cells

Ionizing radiation promotes the induction and accumulation of mutations as a result of DNA damage and inefficient DNA repair. IR deposits energy along specific “tracks” which lead to clustering of DNA lesions (17). The extent of clustering depends on the ionization density and type of radiation, with more clustered damage often observed after exposure to heavy-ion radiation, such as ^56^Fe particles. Such clustered DNA damage caused by high-LET radiation can lead to double strand breaks (DSBs) in DNA and mutations in the absence of proper DNA repair processes (18). Such DSBs can be repaired by non-homologous end-joining (NHEJ) or homologous recombination (HR). The NHEJ pathway seems to play a significant role in DNA repair after exposure to either ^1^H or heavy-ion radiation while HR appears to be more important after heavy-ion radiation (19). Error-prone DNA repair during NHEJ, due to lack of a suitable template, can be a source of mutations post-IR. It should be noted that cells within the bone marrow (BM) often exhibit low levels of expression of many DNA repair proteins, suggesting they may have an inherent inability to repair DNA damage induced by radiation, and therefore are at increased risk of mutations (20). In support of this contention are studies showing that BM cells from mice exposed to 0.5–3 Gy, 1 GeV/n radiation with ^56^Fe particles showed significantly increased chromosomal damage using multi-color FISH techniques (21, 22). ^1^H-IR of 1 Gy, 100 MeV also induced significant DNA damage in mouse BM cells, as assessed by phospho-H2AX foci and multi-color FISH analysis (23, 24).

Exposure of cells to IR can also increase oxidative stress in cells by inducing reactive oxygen or nitrogen species (ROS or RNS), which are the result of interactions between IR and water with other biomolecules in the cell (25). ^1^H-IR of 1 Gy, 150 MeV caused increased oxidative stress as determined by ROS levels and concomitant increases in expression of Nox4 in BM cells (24). ROS and RNS thus generated can interact with DNA and cause more DNA lesions, in addition to those induced by direct DNA damage caused in the radiation tracks. Chronic exposure to oxidative stress can lead to accumulation of such DNA lesions and promote mutagenesis (26). Therefore, the DNA damage and oxidative stress induced in BM by IR, specifically ^1^H- and ^56^Fe-IR, could lead to accumulation of DNA lesions and result in mutations in the hematopoietic stem and progenitor cells.

### Hematopoiesis in Adult Bone Marrow

The BM niche is the predominant site of hematopoiesis and the differentiation of blood cells. This unique microenvironmental niche is also extremely sensitive to low-dose IR exposure (27–29). Disruption of hematopoietic homeostasis can result in hematologic disorders and impact the function of vital organs; for example, abnormalities in hematopoietic cells in the BM can be propagated to the successive blood lineages and result in leukemia. Therefore, it is important to understand the effects of exposure to ^1^H- and ^56^Fe-IR on BM.

Unlike the ablative effect of gamma radiation (γ-IR) on the BM, both short- and long-term effects of particle radiation on this site of hematopoiesis are less understood. Hematopoietic stem cells (HSCs) comprise <0.1% of the BM of adults, yet they produce all of the circulating blood cells that are responsible for constant maintenance and immune protection of the body (28). This exquisitely regulated process known as hematopoiesis occurs in the BM of adults and is responsible for both the maintenance of the primitive HSC and for inducing maturation of these cells to specific blood lineages as the need arises for those particular cell types. Discrete functions performed by the hematopoietic niche may require different growth factors and diverse interactions with different cells types within the site. These various interactions between HSCs and BM stromal cells ensure appropriate cell output to the circulation that change with specific stimuli and demands. Definitive hematopoiesis in the adult BM begins with the differentiation of self-renewing HSCs to hematopoietic multipotent progenitor cells (HPCs or MPPs) (28, 30). These progenitor cells can give rise to the different blood lineages but lack self-renewal capacity. The MPPs develop into committed common lymphoid (CLP) and myeloid (CMP) progenitor cells. The CLP population differentiates into the lymphocyte (NK, B, and T cells) lineages while the CMP gives rise to megakaryocytes, erythrocytes, monocytes, and granulocytes (neutrophils, basophils, and eosinophils). These mature blood cells then exit the BM and enter circulation where they perform important functions. Erythrocytes (red blood cells) are important for oxygen transport, megakaryocytes for blood clotting, and white blood cells (WBCs; namely lymphocytes, monocytes, and granulocytes), function in adaptive and innate immune defenses. Therefore, the process of hematopoiesis in the BM controls the development of all these blood lineages and is responsible for maintaining hematologic homeostasis.

### Effects of ^1^H Radiation on Circulating Blood Cells and Hematopoietic Precursors

Many studies have examined the effects of radiation on circulating blood cells. Irradiation of mice with up to 2 Gy of ^1^H caused significant changes to the peripheral immune cell populations, with different populations exhibiting different sensitivities (31–33). Within the lymphocyte populations, B cells were found to be most sensitive to radiation, followed by T cells and then NK cells which were the most resistant (31). Decreases in WBC populations were dependent on ^1^H-IR dose, but not on dose rate, energy, or fractionation (32, 33). The effects of simulated solar particle events, which are comprised of ^1^H (up to 155 MeV), with a heterogeneous ^1^H dose distribution, also revealed significant reduction (60–90% compared to baseline) in frequencies of circulating WBCs, lymphocytes, neutrophils, monocytes, and eosinophils in both murine and porcine models (34, 35). Murine splenic immune cell populations were impaired at 4 months post-IR with 2 Gy ^1^H, indicating a long-term radiation effect on the precursor hematopoietic populations (36). This was confirmed in recent studies demonstrating that total body irradiation of mice with 1 Gy, 150 MeV of ^1^H caused significant reduction in HSC (Lin^−^c-kit^+^Sca-1^+^) numbers and pluripotency, even at time points as late as 22 weeks after radiation (24). These changes were attributed to the increased levels of oxidative stress in the HSCs, causing increased HSC cell cycling and reduced self-renewal capacity, and resulting in long-term HSC injury. Although ^1^H-IR is a low-LET radiation, its effects on DNA are more damaging than X-rays, indicating the greater capacity to induce changes at the molecular level (37).

### Effects of HZE ^56^Fe Particle Radiation on Circulating Blood Cells and Hematopoietic Precursors

Exposure to HZE particles, such as ^56^Fe, can have even more detrimental effects on BM hematopoietic precursors and mature blood cells. Rats exposed to 1–4 Gy (5 GeV/nucleon) of ^56^Fe-IR had significantly lower counts of circulating leukocytes and monocytes compared to non-irradiated rats for as long as 9 months post-IR (38). Mice irradiated with 6–8 Gy (1 GeV/nucleon) of ^56^Fe particles also showed significantly lower WBC counts 7 days post-IR and lower recovery at 4 weeks post-IR compared to γ-IR mice (39). Examination of the BM revealed extensive cell death, cell cycle arrest and significant selective reduction of myeloid precursor cells in mice exposed to 2–4 Gy of ^56^Fe-IR. Cell cycle arrest of BM cells at the G_1_ phase up to 66 h post-IR was also found in another study with mice irradiated with 1 Gy (1 GeV/nucleon) of ^56^Fe ions (40). Cell cycle arrest corresponded to an increase in cells with ^56^Fe radiation-induced chromosomal aberrations (41). At the molecular level, exposure to 600 MeV, 0.4 Gy ^56^Fe radiation induced DNA hypermethylation in HPCs up to 22 weeks post-IR, suggesting epigenetic reprogramming (42).

Therefore, we *hypothesize* that particle radiation, such as ^1^H and ^56^Fe, which induce profound changes in BM hematopoietic cells, including at the molecular level, may play a significant role in the development of hematological cancers, and thus merits further studies.

## Exposure to ^1^H and ^56^Fe Radiation has Long-Term Effects on Bone Marrow Hematopoietic Multipotent Progenitor Populations

### ^1^H and ^56^Fe Radiation Induced Significant Decrease in Bone Marrow Multipotent Progenitor Cell Numbers

**Figure 1:**
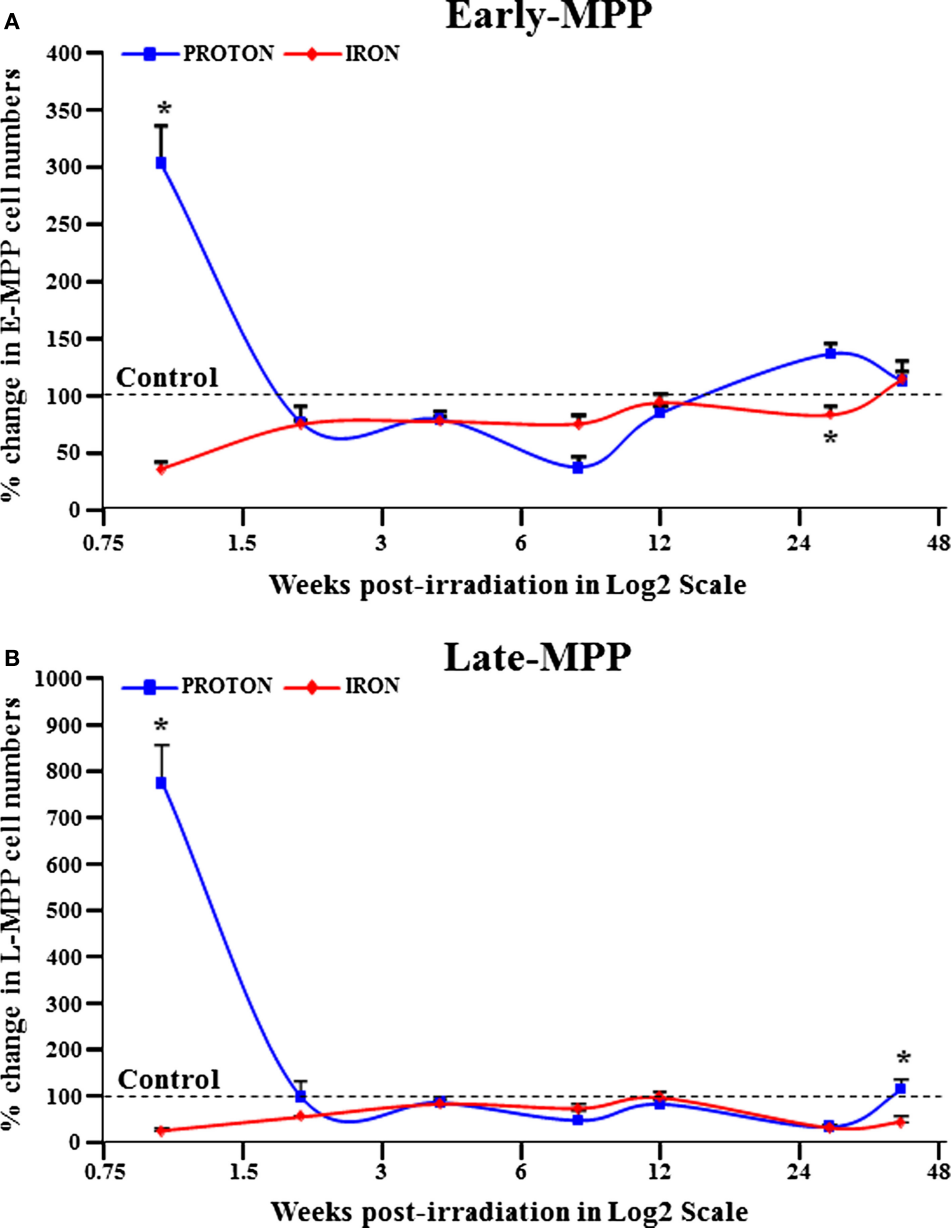
**E-MPP and L-MPP cell numbers are downregulated by ^56^Fe- and ^1^H-IR but recover to control levels by 40 weeks post-IR**. Effect of full-body single dose of proton (^1^H) at 0.5 Gy, 1 GeV and iron (^56^Fe) at 0.15 Gy, 1 GeV/nucleon of ionizing radiation (IR) on survival of multipotent progenitor cell populations was examined. The survival of **(A)** E-MPPs and **(B)** L-MPPs in the BM after particle IR in C57BL/6NT mice were determined at 1, 2, 4, 8, 12, 28, and 40 weeks post-IR. Total BM-derived mononuclear cells were triple-stained with FITC-labeled RAM34 antibody (that consists of CD34, c-kit, and Sca1 antibodies), PE-Cy7-AC133, and PE-hematopoietic lineage cocktail (CD3e, Ly-6G/Ly-6C, CD11b, CD45R/B220, TER-119), then sorted by FASC for **(A)** E-MPPs (CD34^+^/c-kit^+^/Sca-1^+^/AC133^+^/Lin^−^) and **(B)** L-MPPs (CD34^+^/c-kit^+^/Sca-1^+^/AC133^−^/Lin^−^). Percentage changes in cell numbers were calculated relative to control sham irradiated mice, which was set to 100% for each time point. Solid lines represent mean ± SEM (*n* = 6/group) for ^1^H-IR (solid blue lines) and ^56^Fe-IR (solid red lines). “*” represents statistically significant differences compared to control with *p* < 0.05.

To extend our knowledge of the effects of particle radiation on BM hematopoietic populations, whole-body radiation was performed on mice with 0.5 Gy (1 GeV) ^1^H and 0.15 Gy (1 GeV/n) ^56^Fe particles. Fluorescence-activated cell sorting (FACS) was then used to isolate early and late multipotent progenitors (E- and L-MPPs) from BM cells over a time course of 40 weeks post-IR. E-MPPs were defined as Lin^−^/c-kit^+^/Sca1^+^/CD34^+^/AC133^+^ and L-MPPs were Lin-/c-kit^+^/Sca1^+^/CD34^+^/AC133^−^ (43, 44). Compared to control mice, ^1^H-IR caused an initial transient spike in E-MPP and L-MPP cell numbers followed by significant downregulation of these populations at 8 weeks post-IR (Figures 1A,B; Table 1). In contrast, ^56^Fe-IR caused significant loss of E-MPPs and L-MPPs immediately after IR, which was maintained up to 8 weeks post-IR (Figures 1A,B; Table 1). By 40 weeks, the E-MPP and L-MPP populations had recovered and were comparable to control levels for both ^1^H and ^56^Fe radiation (Figures 1A,B). These findings are consistent with the study that showed γ-IR, even at the low dose of 0.4 Gy, was observed to rapidly induce apoptosis in human embryonic stem (ES) cells (45).

**Table 1:** **^56^Fe- and ^1^H-IR resulted in decreased E-MPP and L-MPP cell numbers**.

IR type	Weeks						
	1	2	4	8	12	28	40
**(A) E-MPP**							
^1^H%	665↑*	3↓	13↓	52↓	17↓	66↓	15↑
^56^Fe%	74↓	44↓	16↓	26↓	3↓	69↓	55↓**
**(B) L-MPP**							
^1^H%	203↑*	23↓	21↓	63↓	16↓	36↑***	13↑
^56^Fe%	65↓	25↓	23↓	25↓	6↓	17↓	15↑

*Representation of % change difference in cell number for (A) E-MPPs (CD34^+^/c-kit^+^/Sca-1^+^/AC133^+^/Lin^−^) and (B) L-MPPs (CD34^+^/c-kit^+^/Sca-1^+^/AC133^−^/Lin^−^) from full-body ^1^H-IR and ^56^Fe-IR mice when compared to respective control cell numbers at each time point set at 100%. The arrows show the direction up or down for the population change*.

*  0.001.

**  0.01.

***  0.03.

### ^1^H and ^56^Fe Radiation Significantly Upregulated Expression of Pluripotency Markers in Bone Marrow L-MPPs

**Figure 2:**
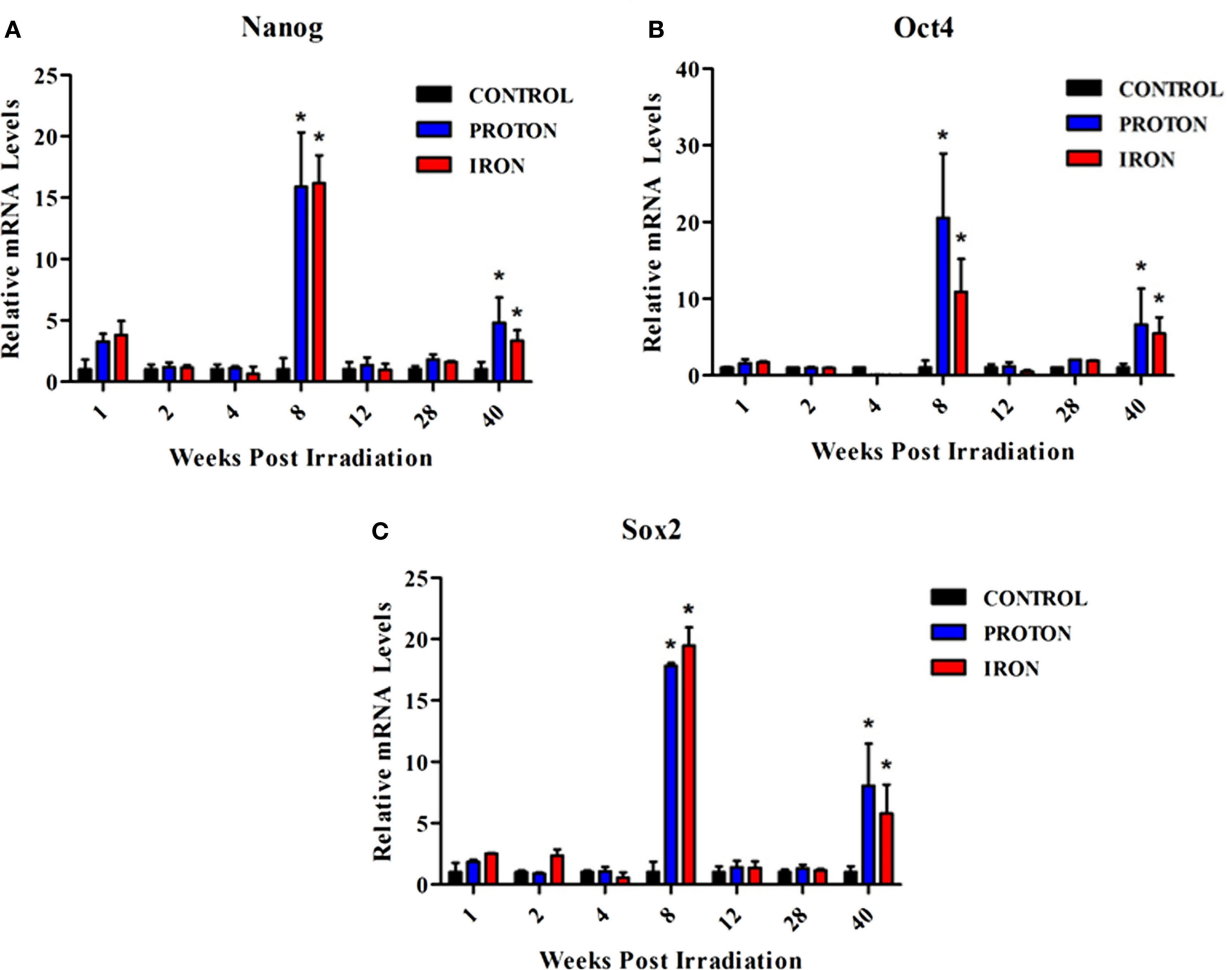
**Expression of pluripotency markers are upregulated in BM-derived L-MPPs post-irradiation with ^1^H or ^56^Fe particles**. After whole-body irradiation with 0.5 Gy, 1 GeV ^1^H and 0.15 Gy, 1 GeV/n ^56^Fe particles, mononuclear cells from bone marrow of C57BL/6NT mice were sorted into L-MPPs (CD34^+^/c-kit^+^/Sca-1^+^/AC133^−^/Lin^−^) by FACS at multiple time points over 40 weeks post-IR. Levels of **(A)** *Nanog*, **(B)** *Oct4*, and **(C)** *Sox2* were analyzed using Taqman probes by qRT-PCR. Relative mRNA levels were calculated with respect to control sham irradiated animals. Bars represent mean ± SEM (*n* = 6/group) for control (solid black bars), ^1^H-IR (solid blue bars), and ^56^Fe-IR (solid red bars). “*” represents statistically significant differences compared to control with *p* < 0.05.

Human ES cells that survived γ-IR exposure exhibited features of pluripotency at 3 weeks post-IR exposure (45). To decipher the molecular events in our radiation study, the expression of pluripotency markers *Sox2*, *Nanog*, and *Oct4* was examined in the L-MPPs for a period of 40 weeks following irradiation with ^1^H or ^56^Fe particles. The qRT-PCR analysis revealed a significant increase in expression of these markers at 8 and 40 weeks after both ^1^H and ^56^Fe irradiation (Figures 2A–C). Of note, it has been shown that ES cells exposed to 3 Gy high-LET carbon ion radiation also maintain their pluripotent state and express Oct3/4 and Sox2; data which agree with our current observations (46). Based on these observations, one could hypothesize that the increase in expression of the pluripotency markers in L-MPPs at 8 weeks post-radiation with ^1^H or ^56^Fe in our study could be the result of preferential expansion of radio-resistant cells. Indeed, this contention is supported by cancer biology studies that have shown a correlation between expression of Oct4 and Sox2 protein and increased resistance of cancer cells to radiotherapy (47, 48). However, the reduced cell numbers we observed at the 8-week time point post-IR (Figure 1B; Table 1) argues against this explanation. An alternative hypothesis to explain our observations is that ^1^H- or ^56^Fe-IR-induced genetic “reprogramming” of the existing L-MPPs. Consistent with this notion, γ-IR was reported to induce reprogramming of cancer stem cells that express the pluripotency genes *Oct4*, *Sox2*, *Nanog*, and *Klf4* in a Notch-dependent manner for up to 5 days post-IR (47, 49). Furthermore, forced expression of *Nanog*, *Oct4*, *Sox2*, and *Lin28* were sufficient to reprogram human somatic cells into pluripotent stem cells (50). Constitutive overexpression of *Nanog* alone is sufficient to promote proliferation of human ES cells while maintaining pluripotency and *Oct4* expression (51, 52). Collectively, our data and previously published data strongly suggest that low doses of ^1^H- or ^56^Fe-IR may induce reprogramming of the L-MPPs to a state of pluripotency while promoting proliferation to replenish the progenitor populations.

### Analysis of L-MPps After Exposure to ^1^H and ^56^Fe Radiation Revealed Distinct Long-Term Genetic Programming

A significant increase in expression of these genes was also observed at 40 weeks post-irradiation with ^1^H and ^56^Fe particles (Figures 2A–C). In order to examine this more closely, a multitude of hematopoiesis-related genes were analyzed in the L-MPPs at the 40-week time point, employing a PCR array for a pilot study (Table 2). Overall, ^1^H- and ^56^Fe-IR induced distinct genetic programs in the L-MPPs, with observable similarities and differences. We found that exposure of L-MPPs to either ^56^Fe- or ^1^H-IR markedly downregulated the expression of several genes that play key functions in the process of hematopoiesis, including *CD164* (sialomucin), which increases adhesion of CD34 + cells to BM stroma and downregulates HPC proliferation (53, 54), and *Fut10*, which can fucosylate selectins for recruitment of progenitors to BM stroma (55, 56) (Table 2). It is possible that downregulation of adhesion molecules could be involved in mobilization of progenitor cells and increase their proliferation. Transcription factors that play an important role in hematopoiesis, such as *Cbfb* and *Ash2l*, were downregulated to a greater extent in L-MPPs exposed to ^56^Fe-IR compared to ^1^H-IR indicating a larger insult by ^56^Fe radiation on BM cells (Table 2) (57, 58). This conclusion is also supported by the observed decrease in expression of immune receptors *TLR3* and *TLR4*, and the co-receptor *CD14* in ^56^Fe-IR L-MPPs, indicating compromised immune responses and immune cell mobilization (Table 2) (59, 60). However, ^1^H-IR L-MPPs showed an increase in expression of these genes, signifying activation of a different epigenetic program. Increased TLR3, TLR4, and CD14 expression on hematopoietic progenitor cells has been correlated with skewing toward myeloid cell differentiation as observed in aging (61, 62). It is possible that the ^1^H- and ^56^Fe-IR may promote the differentiation of these progenitors into the myeloid and lymphoid lineages, respectively. ^1^H-IR exposed L-MPPs showed increased expression of *Notch1* and its downstream target, *Rbpj*. In contrast, L-MPP cells from mice exposed to ^56^Fe-IR showed a discernable decrease in expression of these genes (Table 2). Since activation of Notch1 was shown to promote myeloid differentiation via Rbpj (63), these data may be indicative of myeloid and lymphoid skewing in MPPs induced by ^1^H- and ^56^Fe-IR, respectively. On the other hand, expression of other Notch signaling molecules (*Notch4*, *Jag1*, and *Jag2*) were increased in L-MPPs exposed to ^1^H- and ^56^Fe-IR (Table 2). Interestingly, increased Notch signaling could potentially promote endogenous reprogramming of the cells, as indicated by reports of increased differentiation of cancer stem cells in response to Notch inhibition (64, 65). Therefore, these preliminary gene expression data also supports the possibility of radiation-induced reprogramming of BM progenitors to maintain pluripotency.

**Table 2:** **Exposure to ^1^H or ^56^Fe particles caused notable changes in hematopoietic genes in L-MPPs at 40 weeks post-radiation**.

Group	Gene	Relative mRNA levels in ^1^H-IR L-MPPs	Relative mRNA levels in ^56^Fe-IR L-MPPs
Transcription factors	Cbfb	0.75↓	0.30↓
	Ash21	0.98↓	0.45↓
Adhesion molecules	CD164	0.484↓	0.28↓
	FutlO	0.22↓	0.06↓
Immune receptors	TLR4	2.91↑	0.73↓
	TLR3	12.81↑	0.63↓
	CD14	1.32↑	0.03↓
Notch signaling	Notch1	1.83↑	0.60↓
	Notch4	5.24↑	2.29↑
	Jagl	7.22↑	2.72↑
	Jag2	2.152↑	1.75↑
	Rbpj	1.622↑	0.32↓

*After whole-body irradiation with 0.5 Gy, 1 GeV ^1^H and 0.15 Gy, 1 GeV/n ^56^Fe particles, mononuclear cells from bone marrow of C57BL/6NT mice were sorted into L-MPPs (CD34^+^/c-kit^+^/Sca-1^+^/AC133^−^/Lin^−^) by FACS at 40 weeks post-IR. These experiments were repeated at least twice. Expression of multiple hematopoietic gene transcripts was analyzed using a RT^2^ PCR array. Fold changes were calculated with respect to control sham irradiated animals. The arrows show the direction up or down for the fold change*.

Other studies illustrating radiation-induced endogenous reprogramming have been largely conducted in cancer models. For example, inhibition of Notch signaling partially prevented radiation-induced reprogramming of differentiated breast cancer cells (isolated from patients) into cancer stem cells, thereby preventing their re-acquisition of expression of pluripotency genes *Oct4*, *Nanog*, and *Klf4* (47). High doses of γ-IR was also shown to re-program hepatocellular cancer cell lines to acquire stemness phenotype (49). At the molecular level, radiation can induce epigenetic reprogramming in terms of DNA methylation which can also have important implications in BM progenitor populations (66). Mouse mesenchymal stem cells exposed to non-IR promoted an adipose phenotype (67). Collectively, these observations lend further credibility to our postulation of radiation-induced reprogramming of BM cells, at the molecular level.

## Implications of Radiation-Induced Changes in Bone Marrow Hematopoietic Progenitor Cells

**Figure 3:**
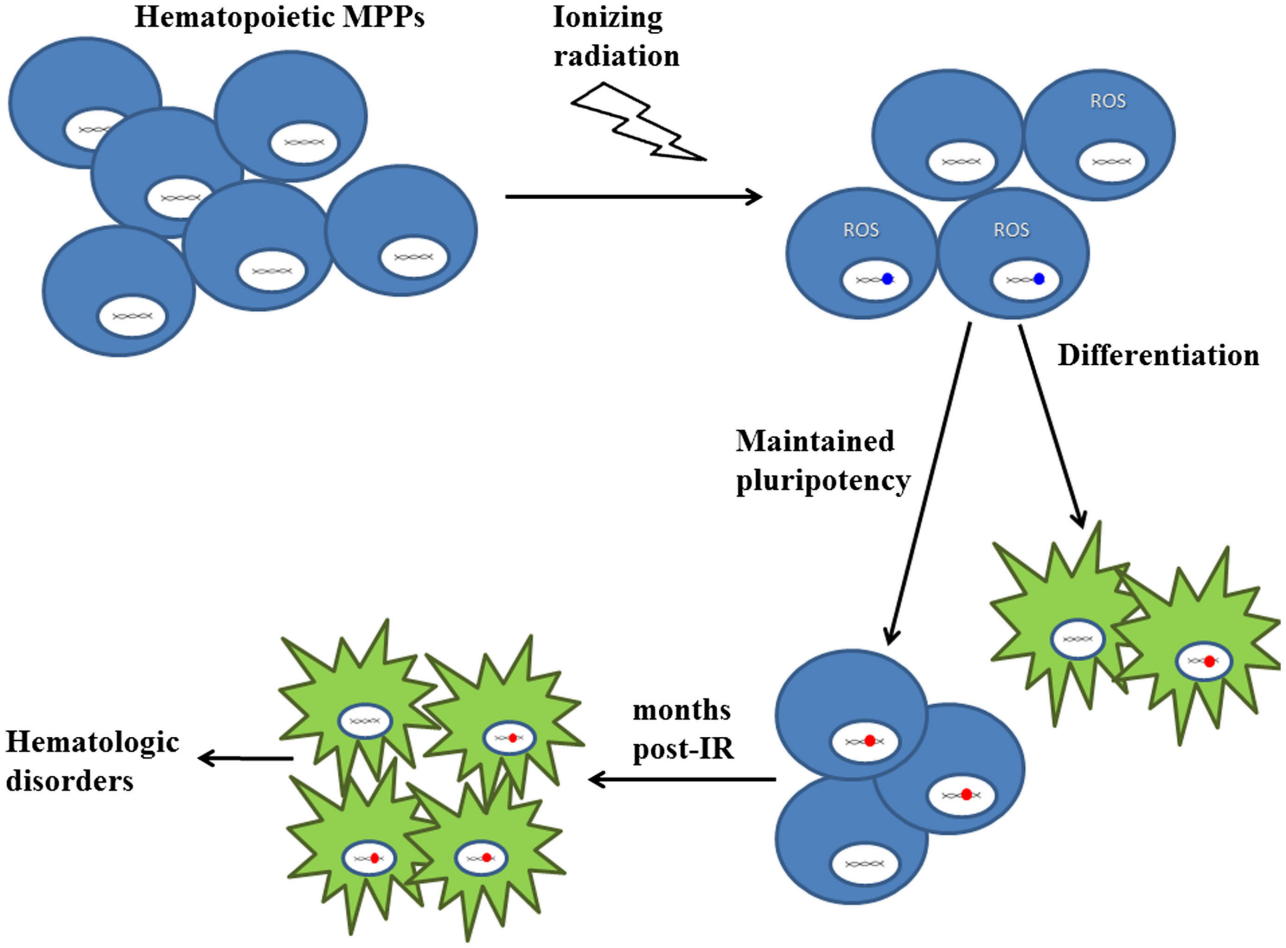
**Proposed model on the effects of low-LET ^1^H and high-LET ^56^Fe radiation on hematopoietic progenitor cells in the bone marrow niche**. There is a significant decrease in MPP population numbers upon exposure to particle radiation. The majority of surviving cells experience DNA damage (depicted in blue) and oxidative stress (depicted by ROS), which could result in DNA mutations (depicted in red). While some MPPs continue to differentiate into blood cells, the remaining cells are “reprogrammed” to maintain pluripotency. As a result, even months post-IR, these “reprogrammed” MPPs [containing IR-induced mutations (depicted in red)] persist and give rise to differentiated blood cells, thereby propagating mutations to subsequent lineages, which may cause hematologic disorders, such as cancer. Blue cells represent multipotent progenitor cells and green cells represent differentiated blood cells.

In our studies into the effects of low-dose low-LET ^1^H and high-LET ^56^Fe-IR on BM hematopoietic progenitor populations, the most striking results were the significant loss of cell numbers and the changes in pluripotent markers in the surviving cells. The long-lasting decrease in the E-MPP and L-MPP populations in the irradiated mice over the course of 40 weeks suggests disrupted hematopoietic homeostasis. Such perturbation of hematopoiesis has the potential to lead to hematological disorders including blood cancers. With regard to the observed genetic changes induced by IR in the surviving L-MPP cell fractions at the 8- and 40-week time point, and supported by the literature reviewed herein, we posit that low-dose IR, especially particle radiation, can induce mutations in the hematopoietic progenitor pools in the BM while concomitantly reprogramming them to a more primitive pluripotent state. While such reprogramming may be beneficial to replenish the progenitor cell pools within the radiation-depleted BM compartments, it may also have severe repercussions on the functions of the subsequent blood cell lineages (Figure 3). One can readily envision the radiation-induced reprogramming of BM progenitor cells, which may also contain radiation-induced mutations, will affect the phenotypes of multiple lymphoid and myeloid cell populations, thereby propagating the mutations to differentiated blood lineages. In particular, the propagation of mutations in oncogenes could promote risk for hematological cancers. It should be noted that high doses of IR are more likely to induce cell apoptosis, which may produce short-term effects, but low-dose radiation can cause significant long-term consequences by inducing mutations that will persist and differentiate into blood cells with altered function. Therefore, exposure to low-dose ^56^Fe or ^1^H particle radiation, as experienced by astronauts in spaceflight or cancer patients that undergo radiation therapy (specifically, the protracted full-body doses), can cause long-term effects in BM cells, thereby increasing their risks of developing (secondary) blood cancers.

## Author Contributions

SM – performed PCR, data analysis, wrote, and edited the manuscript; SS – performed and supervised all experimental studies, analyzed data, and edited the manuscript; MZ – performed PCR and data analysis; KH – reviewed and edited the manuscript; CP – reviewed and edited the manuscript; MC – reviewed and edited the manuscript; KW – reviewed and edited the manuscript; XY – reviewed and edited the manuscript; and DG – conceived the study, designed research, analyzed data, and wrote and edited the manuscript.

## Conflict of Interest Statement

The authors declare that the research was conducted in the absence of any commercial or financial relationships that could be construed as a potential conflict of interest.
