# Corrigendum: Ionizing Particle Radiation as a Modulator of Endogenous Bone Marrow Cell Reprogramming: Implications for Hematological Cancers

**DOI:** 10.3389/fonc.2015.00255

**Published:** 2015-11-25

**Authors:** Sujatha Muralidharan, Sharath P. Sasi, Maria A. Zuriaga, Karen K. Hirschi, Christopher D. Porada, Matthew A. Coleman, Kenneth X. Walsh, Xinhua Yan, David A. Goukassian

**Affiliations:** ^1^Whitaker Cardiovascular Institute, Boston University School of Medicine, Boston, MA, USA; ^2^Cardiovascular Research Center, GeneSys Research Institute, Boston, MA, USA; ^3^Yale Cardiovascular Research Center, Yale School of Medicine, New Haven, CT, USA; ^4^Wake Forest Institute for Regenerative Medicine, Wake Forest School of Medicine, Winston-Salem, NC, USA; ^5^Radiation Oncology, School of Medicine, University of California Davis, Sacramento, CA, USA; ^6^Lawrence Livermore National Laboratory, Livermore, CA, USA; ^7^Tufts University School of Medicine, Boston, MA, USA

**Keywords:** progenitors, endogenous reprogramming, hematological cancer, radiation, HSC

In the paper titled “Ionizing Particle Radiation as a Modulator of Endogenous Bone Marrow Cell Reprogramming: Implications for Hematological Cancers,” there was secretarial error made at our end in “Figure 1,” which should be corrected. At some point of the submission in Figure 1, A and B were disarranged in the slide. No other correction is needed as the text and figure legends are correct.

**Figure 1:**
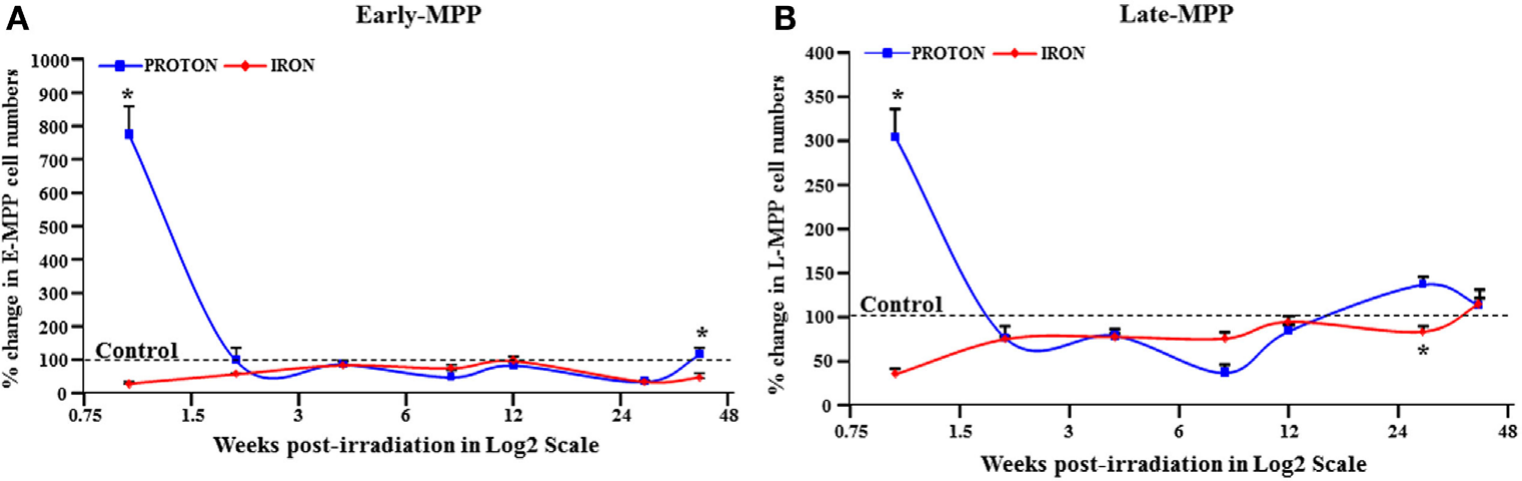
**E-MPP and L-MPP cell numbers are downregulated by ^56^Fe- and ^1^H-IR but recover to control levels by 40 weeks post-IR**. Effect of full-body single dose of proton (^1^H) at 0.5 Gy, 1 GeV and iron (^56^Fe) at 0.15 Gy, 1 GeV/nucleon of ionizing radiation (IR) on survival of multipotent progenitor cell populations was examined. The survival of **(A)** E-MPPs and **(B)** L-MPPs in the BM after particle IR in C57BL/6NT mice were determined at 1, 2, 4, 8, 12, 28, and 40 weeks post-IR. Total BM-derived mononuclear cells were triple-stained with FITC-labeled RAM34 antibody (that consists of CD34, c-kit, and Sca1 antibodies), PE-Cy7-AC133, and PE-hematopoietic lineage cocktail (CD3e, Ly-6G/Ly-6C, CD11b, CD45R/B220, TER-119), then sorted by FASC for **(A)** E-MPPs (CD34^+^/c-kit^+^/Sca-1^+^/AC133^+^/Lin^−^) and **(B)** L-MPPs (CD34^+^/c-kit^+^/Sca-1^+^/AC133^−^/Lin^−^). Percentage changes in cell numbers were calculated relative to control sham irradiated mice, which was set to 100% for each time point. Solid lines represent mean ± SEM (*n* = 6/group) for ^1^H-IR (solid blue lines) and ^56^Fe-IR (solid red lines). “*” represents statistically significant differences compared to control with *p* < 0.05.

## Conflict of Interest Statement

The authors declare that the research was conducted in the absence of any commercial or financial relationships that could be construed as a potential conflict of interest.

